# Ultrasound-guided erector spinae plane block versus thoracic paravertebral block on postoperative analgesia after laparoscopic nephroureterectomy: study protocol of a randomized, double-blinded, non-inferiority design trial

**DOI:** 10.1186/s13063-021-05173-0

**Published:** 2021-04-06

**Authors:** Zhen-Zhen Xu, Xue Li, Zhen Zhang, Zheng-Ye Liu, Lin-Lin Song, Xue-Ying Li, Hong Zhang

**Affiliations:** 1 Department of Anesthesiology and Critical Care Medicine, Peking University First Hospital, No. 8, Xishiku Street, Beijing, 100034 China; 2 Department of Anesthesiology, Civil Aviation General Hospital, Beijing, China; 3 Department of Biostatistics, Peking University First Hospital, Beijing, China

**Keywords:** Erector spinae plane block, Thoracic paravertebral block, Regional anesthesia and analgesia, Pain control, Laparoscopic urological surgery

## Abstract

**Introduction:**

Erector spinae plane block (ESPB) is a novel inter-fascial plane block, which is applied more and more in postoperative pain control, especially in chest surgery. Regional block is advocated in order to decrease opioid consumption and improve analgesia in urological surgery. Therefore, we aimed to explore whether ESPB would have similar analgesia compared with thoracic paravertebral block (TPVB) in laparoscopic nephroureterectomy.

**Methods and analysis:**

This prospective, randomized, double-blinded, non-inferiority trial will enroll 166 patients undergoing laparoscopic nephroureterectomy. Participants will be randomly assigned 1:1 into receiving ESPB or TPVB before surgery. Both ultrasound-guided ESPB and TPVB will be performed with an injection of 0.375% ropivacaine 0.4 ml/kg before anesthesia induction. Standardized patients controlled intravenous analgesia (PCIA) will be applied for each patient. The primary endpoint is the joint of cumulative 24 h opioid (sufentanil) consumption and average pain score via numeric rating scale (NRS) at 24 h after surgery. Secondary endpoints include rescued analgesic demand, cumulative opioid consumption, and pain NRS scores at different preset timepoints within 48 h after surgery. Other predefined outcomes include clinical features of blockage, quality of recovery, subjective sleep quality, time to ambulation and diet, and adverse events, as well as length of stay in hospital and anesthesia cost.

**Discussion:**

Previous studies investigating the analgesic efficacy of ESPB only concentrated on a single endpoint for postoperative pain evaluation, while studies focusing on the direct comparison between ESPB and TPVB in urological surgery are still lacking. Our study is the first trial in non-inferiority design of comparing ESPB and TPVB in patient undergoing laparoscopic nephroureterectomy, and the primary outcome is the joint endpoint of opioid consumption and pain NRS score.

**Trial registration:**

Chinese Clinical Trial Registry ChiCTR 2000031916. Registered on 14 April 2020.

**Supplementary Information:**

The online version contains supplementary material available at 10.1186/s13063-021-05173-0.

## Strengths and limitations of this study

- Our study is the first trial in non-inferiority, double-blinded design of comparing ESPB with TPVB for analgesia in laparoscopic nephroureterectomy.
- The primary outcome in our study is set as a joint endpoint of opioid consumption and pain NRS scores, both of which are deemed equal important in evaluating pain control.
- This study will investigate not only erector spinae plane block efficacy of analgesia, quality of postoperative recovery as usual, but also its clinical features of blockage.
- The relatively small sample size may limit to discriminate the safety consideration between the two blocks technique.
- A single-center trial design may limit its universality.

## Introduction

Upper tract urothelial carcinoma (UTUC) includes carcinoma of the renal pelvis and ureter, as well as bladder. According to the study of European and American population, UTUC accounts for 5–10% of all urothelial carcinoma [1], while this proportion may be higher in China. Laparoscopic nephroureterectomy is increasingly gaining popularity for UTUC therapy due to its advantages of less invasive, faster recovery, and fewer complications. Briefly speaking, this technique is started with detaching the ureter and kidney, and then ligating the renal artery and vein through laparoscopic approach, subsequently resecting the distal ureter, partial bladder, and retrieving all specimens through a 5–8-cm arc or McBurney’s incision in the ipsilateral lower abdomen.

A previous study has shown that patients often suffered moderate to severe pain after laparoscopic nephroureterectomy [2]. Adequate pain control is crucial for postoperative recovery [2]. The traditional analgesia is mainly based on opioids. However, opioids were associated with adverse effects such as respiratory depression, nausea and vomiting, itching, and dizziness, and some patients may be forced to abandon opioids for the reason of intolerance. Thus, multimodal analgesia including trunk block covering the incisions in lateral and anterior abdomen is advocated to enhance early recovery after laparoscopic nephroureterectomy.

Thoracic paravertebral block (TPVB) is a classic trunk block with definite analgesic effect for both somatic and visceral pain. Its efficacy has been demonstrated in urological surgery [3–5].

Erector spinae plane block (ESPB) is a novel inter-fascial plane block first introduced by Forero et al. in 2016 [6], providing wide-ranging analgesia in lung surgery [7–9], laparoscopy [10], mastectomy [11], and pediatric surgery [12, 13]. The proposed mechanism of ESPB is that distribution of local anesthetic solution spreads into the paravertebral space and epidural space [14], which then blocks the dorsal, ventral, and traffic branches of spinal nerve. However, a few studies disagreed [15, 16].

Compared with TPVB, ESPB has the following advantages. First, the dermatomal distribution of sensory loss is extensive, which covers the area ranging from the ipsilateral parasternal to the midline of the lower back in a single shot block. It has been reported that ESPB performed at T5 level can give a sensory loss dermatome from T2 to T9 [6, 17], while local anesthetic solution spreading cranially to the upper thoracic level and caudally as far as the L2-L3 level is founded in ESPB performed at T7 level [18]. Second, regarding to safety, ESPB is conducted in shallow layer and far away from important organs and blood vessels; therefore, the risk of pneumothorax [19, 20], hematoma, nerve injury, and other complications involves less than TPVB theoretically. Finally, when it comes to the simplicity and convenience, the ultrasound imaging features of muscle layers and transverse processes are easy to identify and locate [21].

Although studies have shown that ESPB has a better analgesic effect comparing with traditional opioid intravenous analgesia, there is still a lack of evidence in direct comparing the analgesic efficacy between ESPB and TPVB in urological surgery. Besides, most of previous studies just chose a single endpoint for postoperative pain evaluation and the follow-up period is often limited within 24 h after surgery. Our study is the first trial in non-inferiority design in comparing of ESPB and TPVB with a 48 h follow-up after surgery in patient undergoing laparoscopic nephroureterectomy, and the primary outcome is a joint endpoint of opioid consumption and pain NRS (numeric rating scale) scores.

### Study objectives

The aim of this study is designed to compare ESPB with TPVB in terms of the efficacy of analgesia, the quality of postoperative recovery, and clinical features of blockage in laparoscopic nephroureterectomy. We hypothesize that the analgesic efficacy of ESPB is non-inferior to that of TPVB for patients undergoing laparoscopic nephroureterectomy.

## Methods

### Study design

This trial is a prospective, single-center, two-arm, double-blinded, non-inferiority randomized controlled trial. The study is initiated by Peking University First Hospital, and its design has been completed in strict accordance with the SPIRIT 2013 statement (see Additional file 1 for the SPIRIT Checklist).

**Fig. 1:**
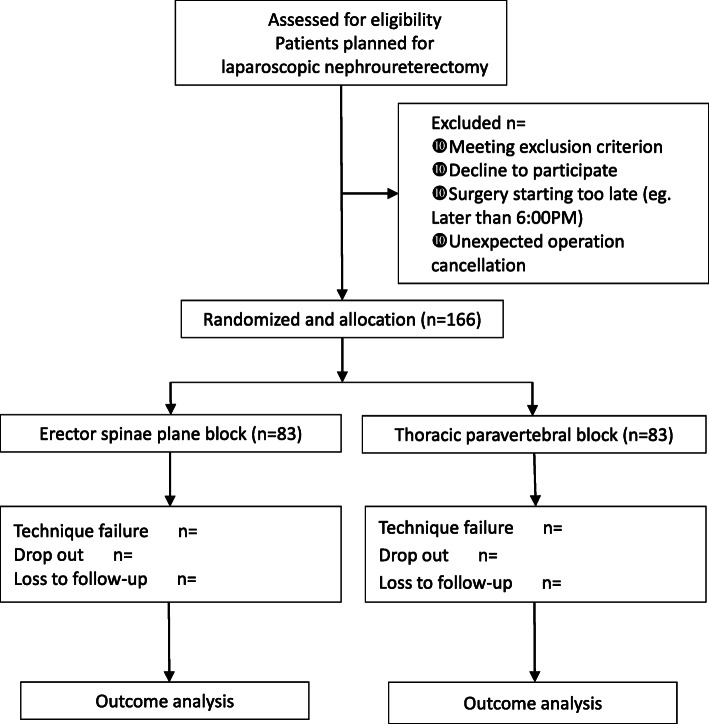
Flowchart of the study

The protocol of this trial has been approved by the Clinical Research Ethics Committee of Peking University First Hospital (registration number: 2019-333) on 29 March 2020 with the latest version 1.1. This trial has been registered on Chinese Clinical Trials registry (identifier: ChiCTR2000031916). We will strictly adhere to the Good Clinical Practice guidelines and the Declaration of Helsinki during the whole period of the study. The flowchart diagram of the study is illustrated in Fig. 1, and the SPIRIT figure of enrolment, interventions, and assessments is presented in Table 1.

**Table 1:**
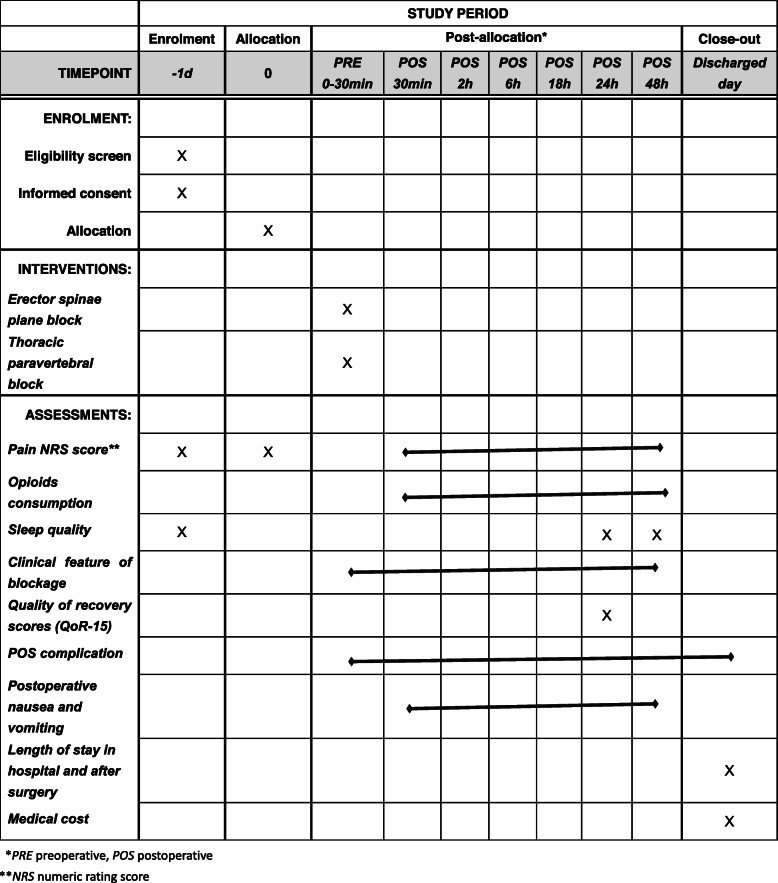
Content and timelines for the schedule of enrolment, interventions, and assessments

*PRE preoperative, *POS* postoperative

**NRS numeric rating score

### Participants

On the day before surgery, patients will be screened by an investigator according to the inclusion/exclusion criteria. If the operation is on Monday, the screening will be performed on the previous Friday. Written informed consent will be obtained from each participant before the enrolment. Patients will be instructed how to self-assess pain severity via the numeric rating scale (NRS, an 11 points scale where 0 = no pain and 10 = most severe pain).

#### Inclusion criteria

Participants who meet all of the following criteria will be enrolled in this study:

1. Between 18 and 75 years of age;
2. Plan to undergo laparoscopic nephroureterectomy;
3. Agree to receive regional nerve block and postoperative intravenous controlled analgesia (PCIA);
4. American Society of Anesthesiologist (ASA) physical status classification I–III.

#### Exclusion criteria

Participants will be excluded if they have any of the following conditions:

1. Refuse to participate in this study;
2. BMI > 35 kg/m^2^ or < 15 kg/m^2^;
3. Combined with severe comorbidities, including but not limiting to renal dysfunction (creatinine > 442 μmol/L or requirement of renal replacement therapy), liver dysfunction (Child-Pugh class C) and heart failure, or ASA physical status classification ≥ IV;
4. Contraindication of deep nerve block, including but not limiting to be allergic to anesthetic drug, coagulation disorder, and infection at the injection site;
5. Chronic opioids dependence or chronic pain over 3 months;
6. Unable to communicate preoperatively due to severe dementia, language barrier, or neuropsychiatric disorder;
7. Unable to perform nerve block procedure due to difficult anatomy through ultrasound scan.

### Randomization, allocation concealment, and blinding

#### Randomization

The SAS 9.3 software package (SAS Institute, Cary, NC, USA) is used to generate random numbers with a block size of 4 by biostatisticians who will not participant in the statistical analysis of the data. The random sequence will be sealed in consecutively numbered opaque envelopes and kept by the study coordinator. Participants will be randomly divided into two groups according to the 1:1 ratio of ESPB group and TPVB group. A research coordinator is designated to distribute and preserve randomization result. The coordinator will open the envelopes for allocation according to the order of enrolment and prepare the study drug.

#### Blinding

The participants will be blinded to the allocation. Because the needle injection of ESPB and TPVB are very close, participants themselves could hardly detect the clinical differences. Anesthesiologists who perform the nerve block and take charge of intraoperative management and surgeons are independent individuals. Only the random sequence number rather than the specific nerve block type will be recorded in the electronic anesthesia information management system (AIMS). Researchers who do not take part in the nerve block and intraoperative management are designated to postoperative follow-up. Besides, trained anesthesiologist who do not perform the block will be designated to evaluate the clinical features of the block objectively. When case report forms (CRFs) are written and checked, the data will be monitored periodically by the Clinical Research Ethics Committee of Peking University First Hospital. After the database is locked, allocation will be handed over and revealed to statistical analysis.

### Intervention

Participants will be admitted to the preoperative preparation area 30 min earlier before transferring to operating room (OR). After establishment of standard monitoring and intravenous access, premedication with 0.02 mg/kg midazolam or 0.08 μg/kg sufentanil will be administrated if necessary. Before regional block, all patients will be positioned lateral decubitus with the operative side up and received ultrasound scan by two experienced anesthesiologists who will not take part in perioperative management and follow-up. After being confirmed as suitable participants for nerve block, patients will receive ultrasound guided ESPB or TPVB block according to the randomized grouping allocation. The drug used for both interventions is the same with a bolus of 0.375% ropivacaine in the volume of 0.4 ml/kg. Once the block is completed, the dermatomes of sensory loss will be assessed by ice-tactus tests every 5 min. Both the onset time (defined as the time to the first detection of a blocked dermatome) and time to fixed sensory block (defined as the time when there was no further extension of the blocked dermatomes) will be recorded. Decreased or loss off thermic sensation of any region will be regarded as successful sensory loss (the illustration for predefined regional distribution was attached in Additional File 2).

#### ESPB group

Patients in ESPB group will be scanned by the linear high-frequency probe firstly placed in sagittal orientation at the middle scapula line. Once the imaging of the 12th rib emerges, T12 spinous process (SP) will be traced by sliding the probe medially and then marked. The probe will be then traced cranially to locate the T10-T11 vertebral. After the same marked method, the probe will be continually moved 3–5 cm laterally and rotated in transverse orientation to identify the muscle layers of erector spinae and transverse processes near the marked site.

After re-confirming the important anatomic structure including lumbar artery under ultrasound scan, a 22-gauge nerve block needle (80 mm, Stimuplex D, B. Braun, Germany) will be inserted in-plane through a medial-lateral direction. Once the needle tip arrives beneath the erector spinae muscle, 3 ml of normal saline will be injected firstly to ensure correct positioning of the needle after aspiration. The prepared study drug will be then injected into this plane with aspiration every 5 ml per injection in case of accidental puncture of vessel or pleura. Successful study drug injection is defined as the appearance of a hypoechoic ellipsoid with well-defined margin beneath erector spinae muscle on ultrasonic view.

#### TPVB group

T10 and T11 vertebral will be located and marked as the same way as that mentioned above in ESPB group. The probe will then be moved 3–5 cm laterally to identify the paravertebral space as the target injection site. After probe being rotated into transverse orientation, a 22-gauge nerve block needle (80 mm, Stimuplex D, B. Braun, Germany) will be inserted using the in-plane technique. Once the needle threads the internal intercostal membrane and arrives in the paravertebral space, 3 ml of normal saline will be injected firstly. If displacement sign of the pleura occurs, the prepared study drug will be then injected into the confirmed paravertebral space. Successful study drug injection is defined as the appearance of pleura displacement sign and hypoechoic ellipsoid in paravertebral space under ultrasonic view.

### Anesthesia management and postoperative analgesia

Intraoperative anesthesia management is performed by an attending anesthesiologist and an assistant; none of whom has knowledge of the allocation. Prednisone methylprednisolone (40 mg) is administered before anesthesia induction, followed by intravenous anesthesia induction with sufentanil (0.1–0.3 μg/kg), propofol (1–2 mg/kg), and rocuronium (0.6 mg/kg). Anesthesia is maintained by intravenous anesthesia combined with or without N_2_O/sevoflurane inhalation to maintain sedation level within the range of 40–60 under bispectral index (BIS) monitor. Opioid analgesics (remifentanil TCI together with sufentanil intermittent administration on demand) are used to maintain analgesia. During the surgery, the fluid volume and infusion speed are adjusted according to hemodynamic monitoring conditions to maintain the blood pressure within 20% of the baseline values. All surgical procedures are performed by fixed surgical team members, and the pneumoperitoneum pressure is often maintained at 12–16 mmHg as usual. After the surgery, standardized patient controlled intravenous analgesia pump is applied for each patient, which was established with 1.25 μg/ml sufentanil and programmed to deliver 4-ml boluses with a lock-out interval of 10 min and no background infusion rate. Patients who are enrolled for this trial will be trained to use a patient controlled intravenous analgesia pump before surgery.

### Endpoints

#### Primary endpoint

The primary outcome in this trial is a joint endpoint of cumulative 24 h opioid consumption after surgery and average pain NRS score at 24 h (valued the average from NRS score at rest and with movement at 24 h) postoperatively.

#### Secondary endpoint

1. Cumulative opioid consumption at other different times (0.5 h, 2 h, 6 h, 18 h, 24 h and 48 h) after surgery, as well as the time interval to the first bolus demand in PCIA pump, the numbers of required and administered boluses in PCIA pump.
2. Somatic and visceral pain NRS scores both at rest and with movement at preset timepoints (0.5th h, 2nd h, 6th h, 18th h, 24th h, and 48th h) postoperatively.
3. Besides PCIA pump, demand of other rescue analgesic (observed at preset different timepoints, including the time interval to the first rescued analgesic in PACU or in ward).

The other endpoints are defined as follows:

1. Clinical features of blockage including time-consumption for the intervention, the onset time and time to fixed sensory block, dermatomal distribution of sensory loss, predefined regional distribution of sensory loss, times of puncture, and the adverse events.
2. Quality of recovery scores (QoR-15) (assessed by QoR-15 questionnaire [22]) face-to-face at 24 h after surgery.
3. Sleep quality scores on the nights of the first 2 days after surgery (assessed with NRS scores, 0 = best sleep quality whereas 10 = worst sleep quality).
4. The incidence of postoperative vomiting and nausea score assessed with NRS scores (0 = no nausea whereas 10 = severe nausea) at the following timepoints (0.5th h, 2nd h, 6th h, 18th h, 24th h, and 48th h) postoperatively.
5. The incidence of postoperative moderate to severe pain.
6. Overall incidence of postoperative complication (observation period: until discharge, classified by Clavien-Dindo Classification) [23].
7. Time to first ambulation and diet (observation from the end of surgery).
8. Length of postoperative hospital stay and total length of hospital stay.
9. Anesthesia cost and total hospitalization cost.

### Safety consideration

We will strictly adhere to the steps to minimize the risk of adverse events which might be caused by regional nerve block. First, we will scan eligible patients before the enrolment and exclude those who are difficult for TPVB and ESPB from anatomical point of view. Both blocks will be performed under directly ultrasound guiding by experienced anesthesiologists. Second, the local anesthetic we used for regional block is 1.5 mg/kg ropivacaine (0.4 ml/kg with 0.375% in our study), which has already been proved to be safe in previous studies [24, 25]. Third, regional block will be performed only after intravenous access and standard monitoring are established; therefore, we can detect any adverse events timely. Lastly, participants will be continuously monitored till 24 h after surgery. Researchers will follow-up the participants at preset timepoints after surgery, and all adverse events occurred during this period will be carefully managed and severe adverse events will be reported to the Clinical Research Ethics Committee as soon as possible. If the patient’s harm level meets the insurance claims, payment will be arranged as soon as possible.

### Data collection and management

Baseline data will be collected, including age, gender, birth date, education years, weight, and height. Co-morbidities as well as important laboratory tests and instrumental examination will be documented. Preoperative recent sleep quality will be assessed with Pittsburgh Sleep Quality Index (PSQI) [26]. Intraoperative data, including duration of anesthesia and surgery, surgical information, and anesthesia medication including opioid consumption and fluid balance will be documented. Pneumoperitoneum pressure will be documented as an average throughout the operation. Outcome data will be evaluated and recorded according to the follow-up plan to ensure all timepoints data are noted.

Based on the original observation records of the participants, the data are collected into the case report forms timely, completely, and correctly. All data will be kept confidentially. Data entry will be performed in a double-input and double-check way with the REDCap (Research Electronic Data Capture) database system developed by the Vanderbilt University. The data will be monitored and sampled regularly in the trial by the Clinical Research Ethics Committee, and the database will be locked after the electronic data is checked. After data entry and verification as required are completed, the case report forms will be filed in numbered order and kept in a specific filing cabinet. We have no plan in interim analysis until the target sample size is achieved.

## Statistical analysis

### Sample size calculation

The primary endpoint of this non-inferiority trial is the joint of cumulative 24 h opioid consumption after surgery and average pain NRS score at 24 h postoperatively.

In a pilot investigation of our patients, the mean (± standard deviation) of the cumulative 24 h opioid (sufentanil) consumption after laparoscopic nephroureterectomy was about 25.7 (± 2.0) μg with TPVB and 35 (± 14.0) μg with non-block respectively (data were unpublished yet). The non-inferiority margin was set as 5 (unit: μg; clinical practice treated it as an acceptable difference) [27]. With a significance level of *α* = 0.05 and a power of 1-β = 90%, the sample size required to detect difference was 77 patients in each group.

A 1–1.3 point difference in pain NRS scores is usually considered as acceptable subjective pain discrimination [28]. When the standard deviation of the pain NRS score was assumed as 2.5 and the non-inferiority margin of NRS was 1 score [29], 70 samples were estimated per group.

Hence, the greater sample calculated above was 77 participants per group. Considering a dropout rate of 5% and technique failure rate of 3%, we finally planned to enroll 83 participants per group (a total of 166 participants). The sample size was estimated by the PASS software (version 11.0; NCSS PASS, UT, USA).

### Endpoints analysis

Baseline characteristic will be compared according the data distribution and type. In addition to graph method for qualitative analysis, Shapiro-Wilk test will be also used for quantitative analysis in review of data distribution. Continuous data with normal or approximate normal distribution will be expressed as mean ± standard deviation, while continuous data with non-normal distribution is expressed as median (interquartile, IQR). Continuous data will be tested by independent sample *t* test or Mann-Whitney *U* test according to data distribution. Categorical data will be expressed as numbers (percentages) and tested by chi-square test/Fisher’s exact test. Time-to-event data will be analyzed by the Kaplan-Meier estimator, with the difference between groups tested by the log-rank method. Two-tailed *p* values of less than 0.05 are regarded as statistically significant. All statistical analyses are performed with the SPSS 25.0 statistical package (IBM SPSS Inc., Chicago, IL, USA).

### Primary endpoint

#### Non-inferiority

Cumulative 24 h opioid consumption and average pain NRS score at 24 h after surgery is the primary joint endpoint, which will be analyzed by “joint hypothesis test.” In this analysis, the intervention group could be considered effective as long as either of the two endpoints is non-inferior. The confidence interval method will be used to perform the non-inferiority test, and the result will be expressed as effect size (difference) (95% CI). If the effect size upper limit of the one-side 95% CI is smaller than 5 in cumulative 24 h opioid consumption, we will conclude that ESPB is non-inferiority to TPVB; in addition, when average pain NRS scores at 24 h after surgery is smaller than 1, ESPB group will be also considered non-inferiority.

#### Superiority

If non-inferiority is conducted for the primary outcome, the superiority of the corresponding comparison will be evaluated for each outcome using an overall *α* of 0.025 with Holm-Bonferroni correction for testing both outcomes (upper limit of 97.5%CI smaller than predefined margin for most significant outcome and upper limit of 95% CI for the other). If superiority is detected on at least either cumulative 24 h opioid consumption or pain NRS scores, the ESPB group will be claimed to be better than TPVB group.

### Secondary endpoint

For the opioid consumption and pain NRS scores at other different timepoints after surgery, repeated measure ANOVA (analysis of variance) will be performed if normality and homogeneity of variance and sphericity hypothesis (Mauchly’s test) are met. If not, one-way ANOVA and its correction (Greenhouse-Geisser coefficient correction and Huynh-Feldt coefficient correction) as well as generalized estimated equation (GEE) model will be performed. As for inter-group comparison, independent sample *t* test will be used for comparison if the distribution accords normality assumption; if distribution is skewed as well as not independent at different timepoints, the difference will be determined by Mann-Whitney *U* test.

Outcome analyses will be performed in the intention-to-treat (ITT) population, i.e., all patients are analyzed in the group to which they are randomized and received at least part of study intervention. A per-protocol (PP) analysis will be also performed for the primary endpoint. Statistical analysis will be performed by professional biostatisticians. All conclusions will be based on the original data.

## Discussion

Clinical anesthesiologists are always looking for a more convenient, effective, and safer analgesic method to control acute pain after surgery. Regional block has become an important role of perioperative multimodal analgesia nowadays. ESPB is thought to be a promising inter-fascial block due to its reliable opiate-sparing effect and easy performing characteristic [30]. Although evidence is increasingly that ESPB is an effective analgesic measure in thoracic surgery, there is still lack of proofs in urological surgery.

Epidural block was recognized as the golden standard analgesia in abdominal surgery in the past decades; however, regional block is mounting to an important position in the area of acute pain management for its convenience and safety. Paravertebral block is deemed as a premium alternative to epidural block and has been used for many years in various kinds of surgery due to its definite analgesia [31]. Most of the recent studies have compared ESPB with intravenous analgesia; however, there is still lack of study comparing ESPB with TPVB except Taketa and colleagues’ work [9]. What is more, the mechanism by which ESPB works through paravertebral space remains conflicting [15]. Therefore, the distribution of sensory loss as well as its analgesic duration also becomes our research interests. Our results will provide new evidence of ESPB into these aspects.

As for pain evaluation, most studies have concentrated on the opioid consumption or pain severity separately, but in fact, these two components contribute equal importance to pain evaluation [32]. Therefore, our research set two endpoints as a joint endpoint to resolve this dilemma. We expect that ESPB with its own merits will be non-inferior to TPVB.

UTUC patients undergoing laparoscopic nephroureterectomy are deemed as a good observation population, due to the incisions of this surgery located both in the lateral and lower abdomen wall and the high incidence of moderate-severe pain after surgery. To our knowledge, this study is the first one to compare ESPB with TPVB in regard to the efficacy and safety in patients undergoing laparoscopic nephroureterectomy. The result might have an influence on enhanced recovery (ERAS) management in urological surgery.

### Trial status

This study is currently at the patient enrolment and data collection stage. The current version of the study protocol is version 1.1 and was approved on 29 March 2020. Patient recruitment started on 27 April 2020 and expected to be finished by 30 May 2022.

## Supplementary Information

**Additional file 1.** SPIRIT 2013 checklist.

**Additional file 2.** Predefined regional distribution.

## Data Availability

The data that support the findings of this study will be available from the corresponding author upon reasonable request.

## Abbreviations

ESPB: Erector spinae plane block
TPVB: Thoracic paravertebral block
ASA: American Society of Anesthesiologist
PCIA: Patient controlled intravenous analgesia
UTUC: Upper tract urothelial carcinoma
RCT: Randomized controlled trial
NRS: Numeric rating scale
BIS: Bispectral index
T: Thoracic
SP: Spinous process
US: Ultrasound
TCI: Targeted controlled infusion
AIMS: Anesthesia intraoperative medical system
CRFs: Case report forms
QoR-15: Quality of recovery 15 questionnaire
PSQI: Pittsburgh sleep quality index
REDCap: Research Electronic Data Capture
ANOVA: Analysis of variance
GEE: Generalized estimated equation
ITT: Intention-to-treat
PP: Per-protocol
ERAS: Enhanced recovery after surgery
