# Supplementary figures and images for: Ultrasound-guided erector spinae plane block versus thoracic paravertebral block on postoperative analgesia after laparoscopic nephroureterectomy: study protocol of a randomized, double-blinded, non-inferiority design trial
Source: Trials. 2021 Apr 6;22:249. doi: 10.1186/s13063-021-05173-0 (PMC8022408; doi:10.1186/s13063-021-05173-0)

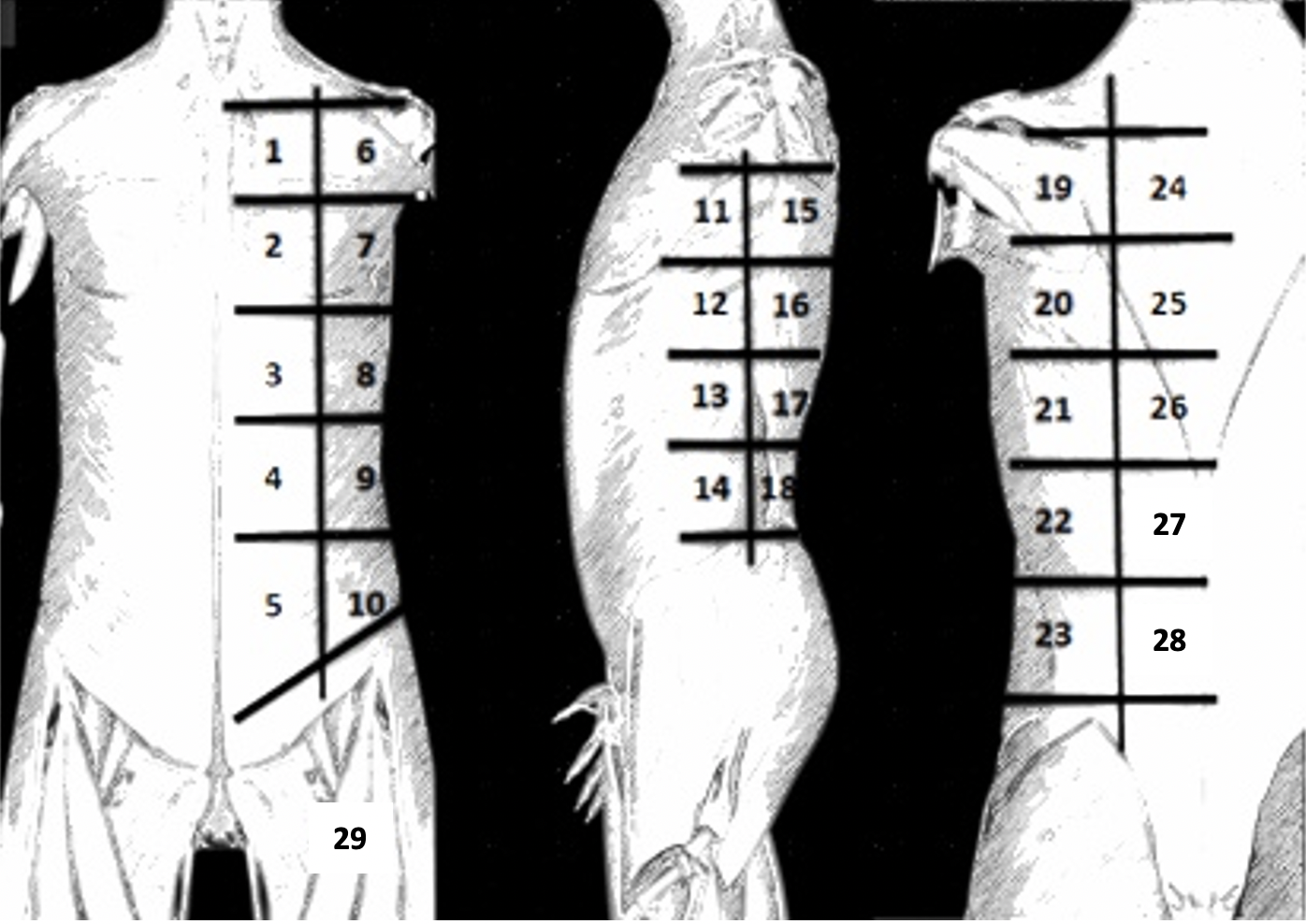

Supplement: Supplementary file 2 — Additional file 2. Predefined regional distribution. [file 13063_2021_5173_MOESM2_ESM.png]
